# Hiperplasia angiolinfoide com eosinofilia: um caso raro em cavidade oral

**DOI:** 10.1590/1677-5449.004516

**Published:** 2016

**Authors:** Jefferson da Rocha Tenório, Amanda Katarinny Goes Gonzaga, Patrícia Guerra Peixe Gonçalves, Denise Hélen Imaculada Pereira de Oliveira, Lélia Maria Guedes Queiroz

**Affiliations:** 1 Universidade Federal do Rio Grande do Norte – UFRN, Departamento de Patologia Oral, Natal, RN, Brasil.

**Keywords:** lesões do sistema vascular, hemangioma, diagnóstico diferencial

## Abstract

A hiperplasia angiolinfoide com eosinofilia (HALE) é considerada uma lesão vascular benigna rara que acomete, principalmente, o tecido cutâneo e subcutâneo da região de cabeça e pescoço, mas incomum na cavidade oral. Sua etiopatogenia permanece indefinida, sendo descrita como proliferação vascular reacional, malformação vascular ou neoplasia. Tem como principal diagnóstico diferencial a doença de Kimura. Este trabalho relata um caso de um paciente do sexo masculino, de 50 anos, que exibia aumento de volume nodular na mucosa do lábio superior, com 3 cm de dimensão e 7 anos de evolução. Após a biópsia excisional, o exame histopatológico mostrou lesão bem encapsulada multilobulada com proliferação de capilares sanguíneos com células endoteliais de aspecto epitelioide, infiltrado inflamatório difuso com linfócitos, plasmócitos, inúmeros eosinófilos e presença de folículos linfoides. A análise imuno-histoquímica revelou positividade para CD34 e Ki-67, o que, juntamente com o exame morfológico, direcionou o diagnóstico para HALE.

## INTRODUÇÃO

Os tumores vasculares compreendem um grupo de lesões cuja característica é seu amplo espectro histopatológico, incluindo lesões como hemangioma, hemangioendotelioma, angiossarcoma e suas variantes epitelioides. Por causa das diferenças em seus comportamentos clínicos e, consequentemente, suas formas de tratamento e prognóstico, é de extrema importância seu acurado e efetivo diagnóstico. Os tumores vasculares epitelioides, por sua vez, são lesões de difícil diagnóstico por causa de sua raridade, suas características morfológicas incomuns e seu comportamento biológico não preditivo^1^.

A hiperplasia angiolinfoide com eosinofilia (HALE) é uma lesão vascular benigna incomum cuja etiologia e patogênese permanecem desconhecidas. A HALE é uma lesão caracterizada histopatologicamente por vasos bem formados, mas frequentemente imaturos, que são delineados por um endotélio de células de formato epitelioide associado a um proeminente infiltrado inflamatório crônico^2^.

O termo HALE foi proposto por Wells e Whimster em 1969. Mais tarde, em 1983, Enzinger e Weiss adotaram o termo hemangioma epitelioide (HE) para essa lesão. Atualmente, a comunidade científica considera tais denominações sinônimos para uma mesma entidade^3^.

A HALE pode acometer qualquer região da cabeça e do pescoço, incluindo ossos maxilares, glândulas salivares, tecidos musculares e pele. A mucosa oral é um sítio pouco frequente e com escassos relatos na literatura, mas, quando ocorre, os locais mais afetados são os lábios, a mucosa jugal e a língua^4^.

O objetivo deste manuscrito é relatar um caso raro de HALE em mucosa labial e, adicionalmente, discutir os principais critérios de diagnóstico histopatológicos e imuno-histoquímicos.

## DESCRIÇÃO DO CASO

**Figura 1:**
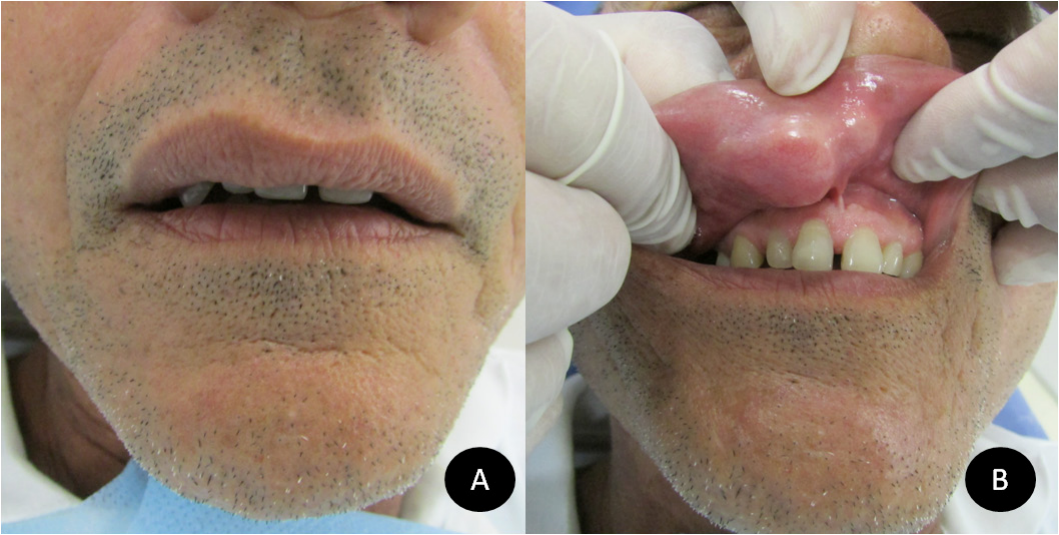
Exame clínico: (A) Discreto aumento de volume na região labial superior; (B) Notável lesão nodular na região de submucosa labial com superfície de coloração semelhante à da mucosa oral.

Paciente do sexo masculino, 50 anos, cor parda, procurou o Serviço de Diagnóstico Oral da Universidade Federal do Rio Grande do Norte (UFRN), Natal, RN, Brasil, com queixa de lesão assintomática em mucosa labial superior com cerca de 7 anos de evolução. A história médica não era contribuitória. O exame extraoral revelou discreto aumento de volume na região subnasal (Figura 1A). Os linfonodos cervicais não estavam palpáveis. O paciente não relatou história de trauma local ou picada de insetos. O exame intraoral revelou nódulo localizado no plano submucoso na mucosa labial superior, firme à palpação, com aspecto tumoral e cor semelhantes à mucosa, medindo aproximadamente 2,5 cm (Figura 1B). A hipótese diagnóstica foi a de neoplasia benigna de glândula salivar. Foi realizada biópsia excisional sob anestesia local, sendo obtidos cinco pequenos fragmentos de tecido mole, que foram fixados em formol a 10% e enviados para análise histopatológica.

**Figura 2:**
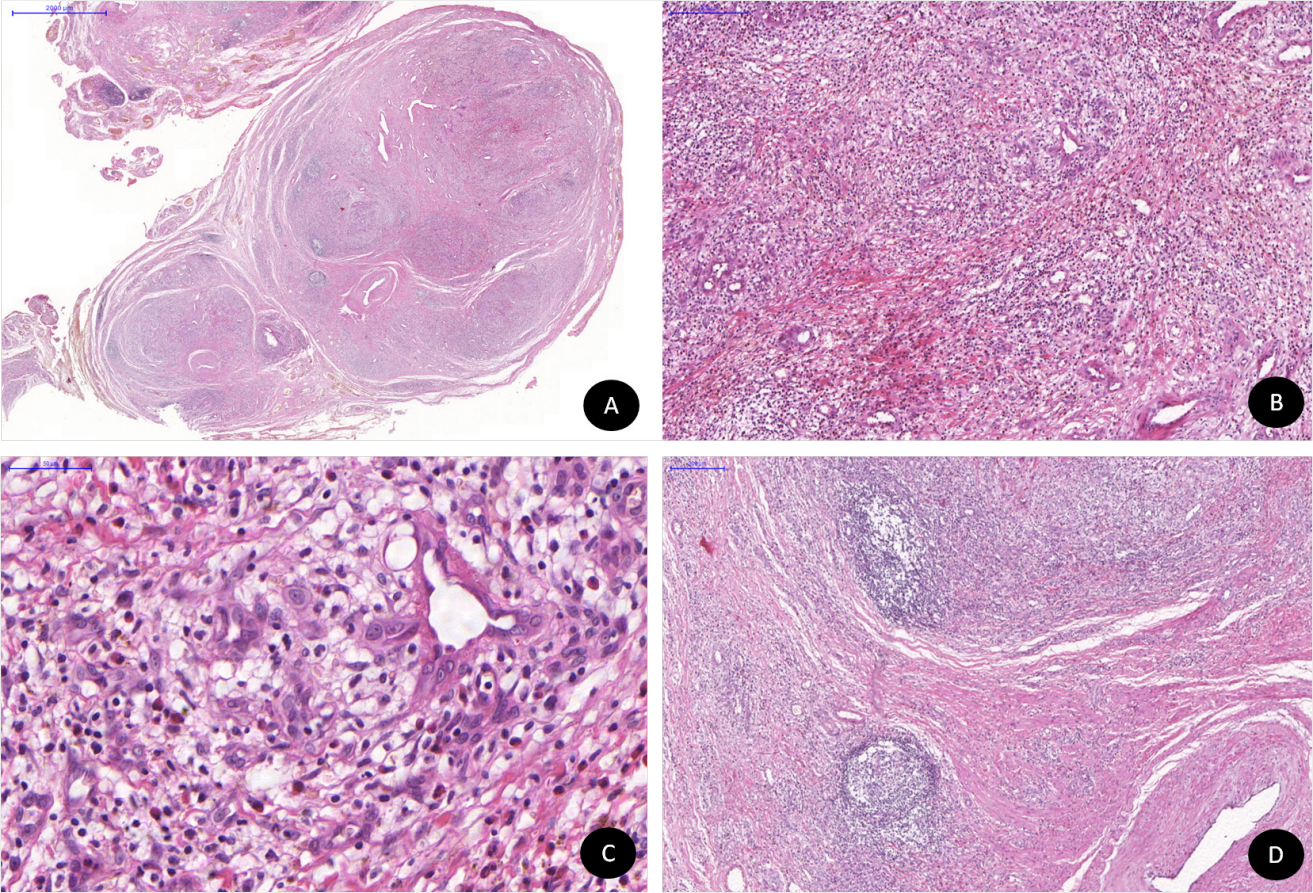
Exame histopatológico: (A) Lesão de aspecto multinodular; (B) Proliferação vascular em arranjo lobular separada por finos septos de tecido conjuntivo fibroso e permeada por infiltrado inflamatório misto; (C) Pequenos vasos com endotélio de células epitelioides e inúmeros eosinófilos adjacentes às formações vasculares; (D) Centros germinativos.

O exame histopatológico revelou uma lesão vascular benigna, caracterizada pela proliferação de capilares sanguíneos com espessa parede vascular, além de expressiva quantidade de eosinófilos. Tratava-se de uma lesão bem delimitada e encapsulada com múltiplos lóbulos separados por septos de tecido conjuntivo fibroso (Figuras 2A e 2B). Os capilares eram delineados por células endoteliais de aparência epitelioide, com citoplasma amplo e eosinofílico, além de núcleo vesicular e nucléolos evidentes (Figura 2C). Áreas de hialinização perivascular também foram observadas. A lesão possuía evidente componente inflamatório, composto principalmente por eosinófilos, linfócitos e plasmócitos (Figura 2C). Alguns centros germinativos também compunham a lesão (Figura 2D). Atipias celulares e mitoses não foram encontradas.

**Figura 3:**
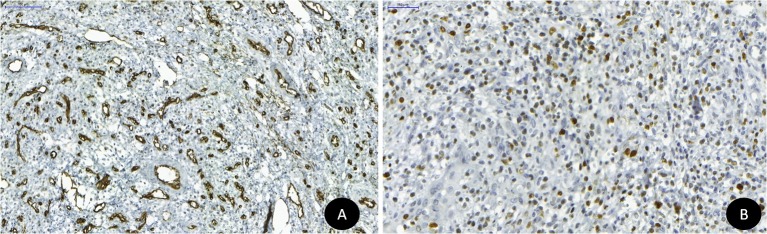
Análise imuno-histoquímica: (A) Padrão de marcação para a proteína CD34; (B) Padrão de marcação para a proteína Ki-67.

O espécime foi então submetido a cortes de 3 μm para estudo imuno-histoquímico com os anticorpos anti-CD-34 e Ki-67. A lesão revelou moderada (15-50% de células marcadas sob aumento de 200x) positividade para o marcador CD-34 nas estruturas vasculares (Figura 3A), além de moderada marcação para o Ki-67, o que reafirmou o potencial proliferativo dessa lesão (Figura 3B).

Com base nos dados clínicos, histopatológicos e imuno-histoquímicos, o diagnóstico final foi de HALE. O paciente continua sob acompanhamento clínico e, até o presente momento, não apresentou recidiva.

## DISCUSSÃO

A HALE em mucosa oral é uma doença rara frequentemente confundida com outras lesões vasculares de aparência epitelioide e com outras lesões de tecidos moles de natureza não vascular. Na literatura, vários outros termos têm sido utilizados pelos mais diversos pesquisadores para descrever essa lesão, entre eles: hemangioma epitelioide, hiperplasia linfoide angioblástica nodular com eosinofilia, linfofoliculose, hemangioma histiocitoide, granuloma pseudopiogênico e granuloma piogênico atípico^5^.

A etiologia da HALE ainda não foi esclarecida, mas foi proposto que sua patogênese inclui processo neoplásico, reação de hipersensibilidade, reação vascular inflamatória ou reação tecidual a trauma prévio, como visto em casos de fístulas arteriovenosas traumáticas adquiridas. Também é relatado que elevados níveis séricos de estrogênio (como ocorre na gravidez ou no uso de contraceptivos orais) podem promover o crescimento dessa lesão. No entanto, atualmente, a hipótese mais aceita é a de que se trata de uma hiperplasia vascular reacional ocasionada por diferentes estímulos^6^.

Muitos casos de HALE são relatados por surgirem de um trauma prévio, o que não aconteceu no presente relato.

Clinicamente, apresenta-se como pápulas ou nódulos menores que 3 cm, de coloração rosa a acastanhada e superfície lisa, que acometem, principalmente, a superfície da pele e o tecido subcutâneo da cabeça e do pescoço. As regiões de cabeça e pescoço mais acometidas são orelha e couro cabeludo^3^. Casos em cavidade oral são raros^4^. Prurido, dor, ulceração e sangramento podem estar associados^3^^,^ 4, embora não tenham sido vistos no presente caso.

É mais comum entre a segunda e a quarta décadas de vida, com predomínio entre o sexo feminino. Pode se apresentar de forma solitária, como no presente caso, ou de forma múltipla e disseminada^7^.

A HALE é também chamada de hemangioma epitelioide devido à presença de células endoteliais arredondadas com citoplasma abundante. Essas células circundam os numerosos espaços vasculares presentes na lesão. Histopatologicamente, observa-se também um significativo componente inflamatório composto por linfócitos, plasmócitos e numerosos eosinófilos^8^. A ausência de eosinófilos na lesão dificulta o diagnóstico, o que não ocorreu no presente caso.

Os principais diagnósticos diferenciais, do ponto de vista clínico, são: tumores de glândulas salivares, hemangioma, sarcoma de Kaposi, linfoma, picada de insetos e granuloma piogênico. Quando observada histopatologicamente, a HALE se assemelha a lesões vasculares de aparência epitelioide, como as variantes epitelioides do hemangioendotelioma e do angiossarcoma. Contudo, o hemangioendotelioma epitelioide é uma lesão *borderline* caracterizada por moderado pleomorfismo celular e canais vasculares fracamente definidos. Além disso, as células epitelioides dessa lesão apresentam vacuolização intracitoplasmática característica. Por sua vez, a variante epitelioide do angiossarcoma é uma lesão de comportamento clínico agressivo, infiltrativo e destrutivo, com elevado pleomorfismo celular, além da presença de mitoses e áreas de necrose^9^.

O principal diagnóstico diferencial da HALE é a doença de Kimura – ambas são consideradas dermatoses eosinofílicas e apresentam semelhanças morfológicas. No entanto, a doença de Kimura é marcada por eosinofilia sérica e hiperimunoglobulinemia, além de envolvimento linfonodal^9^^,^ 10. No paciente do presente relato, não foram encontradas características relacionadas à doença de Kimura.

A análise imuno-histoquímica revela positividade para CD31, CD34 e fator VIII^11^. No presente caso, houve imunopositividade para CD-34, o que corrobora os achados da literatura.

Essa lesão pode ser tratada através de injeção intralesional de isotretinoína, glicocorticoides, interferon alfa e terapia de irradiação. No entanto, a excisão cirúrgica é considerada a terapia mais eficaz^12^, realizada no caso do presente relato. Alguns autores relatam taxas de recidiva de até 2/3 dos casos, porém, neste caso, o paciente está sob acompanhamento há seis meses sem recidiva da lesão. O prognóstico, após a ressecção cirúrgica, é favorável. O curso da lesão é crônico e benigno, e não há relatos de transformação maligna^13^.
