# Supplementary material for: Hiperplasia angiolinfoide com eosinofilia: um caso raro em cavidade oral
Source: J Vasc Bras. 2016 Oct-Dec;15(4):317–21. [Article in Portuguese] doi: 10.1590/1677-5449.004516 (PMC5829732; doi:10.1590/1677-5449.004516)
Supplement: Translation

# Translation: Angiolymphoid hyperplasia with eosinophilia: a rare case in the oral cavity

**Authors:** Jefferson da Rocha Tenório, Amanda Katarinny Goes Gonzaga, Patrícia Guerra Peixe Gonçalves, Denise Hélen Imaculada Pereira de Oliveira, Lélia Maria Guedes Queiroz

**Affiliations:** 1 Universidade Federal do Rio Grande do Norte – UFRN, Departamento de Patologia Oral, Natal, RN, Brazil.

**Keywords:** vascular system lesions, hemangioma, differential diagnosis

## Abstract

Angiolymphoid Hyperplasia with eosinophilia (ALHE) is considered a rare, benign vascular lesion that mainly affects the skin and subcutaneous tissues of the head and neck, but is uncommon in the oral cavity. Its etiology remains unclear and it has been described as a reactive vascular proliferation, vascular malformation or neoplasm. Kimura's disease is the primary entity to consider in differential diagnosis. Here we report on a rare case of ALHE involving the upper lip of a 50-year-old male patient that had a nodular swelling with approximately 3 cm, 7 years after initial onset. An excisional biopsy was performed and histopathologic examination revealed a well-encapsulated, multi-lobed lesion with proliferation of blood capillaries, displaying endothelial cells of epithelioid appearance, diffuse inflammatory infiltrate with lymphocytes, plasma cells, numerous eosinophils, and presence of lymphoid follicles. Immunohistochemical tests were positive for the markers CD34 and Ki-67 that, in combination with the results of morphological examination, were suggestive of a diagnosis of ALHE.

## INTRODUCTION

As a group, the primary characteristic of vascular tumors is their wide spectrum of histopathological types, including lesions such as hemangioma, hemangioendothelioma, angiosarcoma, and their epithelioid variants. Accurate and definitive diagnosis is extremely important because of the differences in their clinical behavior and, consequently, their treatments and prognoses. In turn, epithelioid vascular tumors are difficult to diagnose because of their rarity, their uncommon morphological characteristics and their unpredictable biological behavior.^1^

Angiolymphoid hyperplasia with eosinophilia (ALHE) is an uncommon benign vascular lesion with etiology and pathogenesis that are as yet unknown. Histopathologically, ALHE is characterized by well-formed vessels, that are frequently immature, delineated by an endothelium of epithelioid cells associated with abundant chronic inflammatory infiltrate.^2^

The term ALHE was proposed by Wells and Whimster in 1969. More recently, in 1983, Enzinger and Weiss adopted the term epithelioid hemangioma (HE) to describe this lesion. Nowadays the scientific community considers both terms to be synonyms for a single entity.^3^

Angiolymphoid hyperplasia with eosinophilia can involve any part of the head and neck, including maxillary bones, salivary glands, muscle tissues and skin. The oral mucosa is an uncommon target and there are few reports in the literature, but, when it is involved, the most often affected sites are the lips, the buccal mucosa, and the tongue.^4^

The objective of this paper is to report a rare case of ALHE of the mucosa of the upper lip and, additionally, to discuss the most important criteria for histopathological and immunohistochemical diagnosis.

## CASE DESCRIPTION

A 50-year-old, brown-skinned, male patient presented at an Oral Diagnosis Service run by the Universidade Federal do Rio Grande do Norte (UFRN), Natal, RN, Brazil, complaining of an asymptomatic lesion on the mucosa of the upper lip with onset around 7 years previously. His medical history did not contribute to diagnosis. Extraoral examination revealed a discrete increase in volume in the area below the nose (Figure 1A). Cervical lymph nodes were not palpable. The patient did not report any previous local trauma or insect bites. Intraoral examination revealed a nodule located on the submucosal plane of the mucosa of the upper lip that was firm to the touch, with a tumoral appearance and color similar to the mucosa, measuring approximately 2.5 cm (Figure 1B). The initial diagnostic hypothesis was benign salivary gland neoplasm. An excisional biopsy was performed under local anesthesia and five small fragments of soft tissue were removed, fixed in 10% formol and sent for histopathological analysis.

**Figure 1:** Clinical examination: (A) Discrete increase in volume in the upper lip area; (B) Evident nodular lesion in the submucosa of the upper lip, with a surface color similar to the oral mucosa.

The histopathological results indicated a benign vascular lesion characterized by proliferation of blood capillaries, with thick vascular walls, and a considerable quantity of eosinophils. The lesion was well-delineated and encapsulated with multiple lobes separated by septa of fibrous connective tissue (Figures 2A and 2B). The capillaries were delineated by endothelial cells with an epithelioid appearance, large and eosinophilic cytoplasm, and vesicular nuclei with visible nucleoli (Figure 2C). Areas of perivascular hyalinization were also observed. The lesion had an evidently inflammatory component, primarily comprising eosinophils, lymphocytes, and plasmacytes (Figure 2C). The lesion also contained a number of germinal centers (Figure 2D). No cell atypia or mitoses were detected.

**Figure 2:** Histopathological examination: (A) Lesion with a multi-nodular appearance; (B) Vascular proliferation in a lobular arrangement separated by fine septa of fibrous connective tissue and permeated by mixed inflammatory infiltrate; (C) Small vessels with endothelium of epithelioid cells and countless eosinophils adjacent to the vascular formations; (D) Germinal centers.

The specimen was then cut into 3 μm slices for immunohistochemical tests using anti-CD-34 and Ki-67 antibodies. The lesion was moderately positive (15-50% of cells marked at 200x magnification) for the CD-34 marker in vascular structures (Figure 3A), and moderate for the Ki-67 marker, reaffirming the lesion’s proliferative potential (Figure 3B).

**Figure 3:** Immunohistochemical analysis: (A) Pattern of marking for CD34 protein; (B) Pattern of marking for Ki-67 protein.

Based on clinical, histopathological, and immunohistochemical data, the final diagnosis was ALHE. The patient is still in clinical follow-up and, to date, has not suffered a relapse.

## DISCUSSION

Angiolymphoid hyperplasia with eosinophilia of the oral mucosa is a rare disease that is often confused with other vascular lesions with an epithelioid appearance and with other soft tissues lesions of a non-vascular nature. Many other terms have been used in the literature by a large number of different researchers to describe this lesion, including: epithelioid hemangioma, nodular angioblastic lymphoid hyperplasia with eosinophilia, lymphofolliculosis, histiocytoid hemangioma, pseudopyogenic granuloma and atypical pyogenic granuloma.^5^

The etiology of ALHE has not yet been elucidated, but it has been proposed that its pathogenesis includes neoplastic processes, hypersensitivity reactions, vascular inflammatory reactions, or tissue reactions to a prior trauma, as is seen in cases of acquired traumatic arteriovenous fistulas. It has also been reported that elevated serum estrogen (as occur during pregnancy or when taking oral contraceptives) can provoke growth of this lesion. However, the most widely accepted hypothesis is that the lesion is a reactive vascular hyperplasia caused by several different stimuli.^6^

Many cases of ALHE are reported to have emerged after a previous trauma, but that was not the case in this report.

Clinically, ALHE presents as papules or nodules smaller than 3 cm, with a color ranging from pink to brown and a smooth surface, that primarily involve the surface of the skin and subcutaneous tissues of the head and neck. The areas of the head and neck most affected are the ears and scalp.^3^ Cases in the oral cavity are rare.^4^ Itching, pain, ulceration, and bleeding may all be present,^3^^,^ 4 although they were not observed in the case described here.

The condition is most common between the second and fourth decades of life and has a predilection for women. The lesions may be solitary, as in the present case, or they may be multiple and disseminated.^7^

Angiolymphoid hyperplasia with eosinophilia is also known as epithelioid hemangioma because of the presence of rounded endothelial cells with abundant cytoplasm. These cells surround the numerous vascular spaces within the lesion. Histopathologically, there is also a significant inflammatory component composed of lymphocytes, plasma cells, and copious eosinophils.^8^ Absence of eosinophils in the lesion makes diagnosis more difficult, but this was not the case here.

From a clinical point of view, the major alternatives to be ruled out in differential diagnostic are: salivary gland tumors, hemangioma, Kaposi’s sarcoma, lymphoma, insect bites, and pyogenic granuloma. Histopathologically, ALHE is similar in appearance to epithelioid vascular lesions, such as epithelioid variants of hemangioendothelioma and angiosarcoma. Notwithstanding, epithelioid hemangioendothelioma is a borderline lesion characterized by moderate cellular pleomorphism and weakly defined vascular channels. Additionally, the epithelioid cells of this lesion exhibit characteristic intracytoplasmic vacuolization. In turn, the epithelioid variant of angiosarcoma is a lesion with aggressive clinical behavior, that is infiltrative and destructive, with elevated cellular pleomorphism, in addition to exhibiting presence of mitoses and areas of necrosis.^9^

Kimura's disease is the primary entity to consider in differential diagnosis, since both are considered eosinophilic dermatosis and they have morphological similarities. However, Kimura’s disease is marked by serum eosinophilia and hyperimmunoglobulinemia and there is lymph node involvement.^9^^,^ 10 In the patient described here, characteristics related to Kimura’s disease were not present.

Immunohistochemical analysis results are positive for CD31, CD34 and factor VIII.^11^ In the present case, there was immunopositivity for CD-34, which agrees with findings in the literature.

This type of lesion can also be treated with intralesional injection of isotretinoin, glucocorticoids, interferon alpha, and radiotherapy. However, surgical excision is considered the most effective treatment,^12^ and this was carried out in the present case. Some authors report relapse rates of up to 2/3 of cases, but this patient has been in follow-up for 6 months with no relapse of the lesion. Prognosis is favorable after surgical resection. The lesion has a chronic and benign course and there are no reports of malignant transformation.^13^
